# Herpes simplex virus induces neural oxidative damage via microglial cell Toll-like receptor-2

**DOI:** 10.1186/1742-2094-7-35

**Published:** 2010-06-28

**Authors:** Scott J Schachtele, Shuxian Hu, Morgan R Little, James R Lokensgard

**Affiliations:** 1 Center for Infectious Diseases and Microbiology Translational Research, Department of Medicine, University of Minnesota, MN, USA

## Abstract

**Background:**

Using a murine model of herpes simplex virus (HSV)-1 encephalitis, our laboratory has determined that induction of proinflammatory mediators in response to viral infection is largely mediated through a Toll-like receptor-2 (TLR2)-dependent mechanism. Published studies have shown that, like other inflammatory mediators, reactive oxygen species (ROS) are generated during viral brain infection. It is increasingly clear that ROS are responsible for facilitating secondary tissue damage during central nervous system infection and may contribute to neurotoxicity associated with herpes encephalitis.

**Methods:**

Purified microglial cell and mixed neural cell cultures were prepared from C57B/6 and TLR2^-/- ^mice. Intracellular ROS production in cultured murine microglia was measured via 2', 7'-Dichlorofluorescin diacetate (DCFH-DA) oxidation. An assay for 8-isoprostane, a marker of lipid peroxidation, was utilized to measure free radical-associated cellular damage. Mixed neural cultures obtained from β-actin promoter-luciferase transgenic mice were used to detect neurotoxicity induced by HSV-infected microglia.

**Results:**

Stimulation with HSV-1 elevated intracellular ROS in wild-type microglial cell cultures, while TLR2^-/- ^microglia displayed delayed and attenuated ROS production following viral infection. HSV-infected TLR2^-/- ^microglia produced less neuronal oxidative damage to mixed neural cell cultures in comparison to HSV-infected wild-type microglia. Further, HSV-infected TLR2^-/- ^microglia were found to be less cytotoxic to cultured neurons compared to HSV-infected wild-type microglia. These effects were associated with decreased activation of p38 MAPK and p42/p44 ERK in TLR2^-/- ^mice.

**Conclusions:**

These studies demonstrate the importance of microglial cell TLR2 in inducing oxidative stress and neuronal damage in response to viral infection.

## Background

Exposure to an invading pathogen initially triggers robust innate immune responses. These responses involve recruitment and activation of phagocytic macrophages, neutrophils and, during central nervous system infection, the activation of resident microglia. One effective method that immune cells utilize to eliminate invading pathogens is through the rapid and robust production of reactive oxygen species (ROS), termed the respiratory burst, which facilitates damage to the invading pathogen. Some of the reactive species generated include superoxide (O2^-^), hydroxyl radical (OH), hydrogen peroxide (H_2_O_2_), hypochlorite (OCl^-^), and peroxynitrite (OONO^-^). ROS production, while beneficial in clearing invading pathogens, can also cause irreparable harm through bystander damage to crucial host cells.

Herpes simplex virus (HSV)-1 infection of the brain results in devastating necrotizing encephalitis. Using a murine model of HSV-1 encephalitis our laboratory has shown that intranasal delivery of HSV induces a robust activation of brain microglial cells, production of proinflammatory mediators, and focal tissue damage which, if left untreated, can result in prolonged brain inflammation, and compromised brain function or death [1-5]. It is becoming increasingly clear that reactive species are responsible for mediating many of the secondary mechanisms of tissue damage during and subsequent to viral brain infection, including herpes encephalitis [6].

Microglial cell production of ROS is implicated in neurototoxicity associated with HIV-associated dementia, Alzheimer's disease, Parkinson's disease and Amyotrophic lateral sclerosis [7,8]. While *in vitro *studies in our laboratory implicate microglial cells as the primary mediator of proinflammatory cytokines and chemokines during HSV-1 infection, little is known about ROS production and its impact on the brain during viral encephalitis. While reactive species themselves are short lived and difficult to measure, detection of secondary products such as lipid peroxidation (F2-isoprotanes and F4-neuroprostanes), tyrosine nitrosylation and DNA-oxidation (8-hydroxydeoxyguanosine) are useful indicators of oxidative damage *in vivo*. Indeed, our laboratory has detected increases in 8-isoprostane, 8-hydroxydeoxyguanosine and 3-nitrotyrosine during active experimental herpes encephalitis [3]. Radical-induced tissue injury has also been detected during latent herpes infection [6]. These studies indicate that the cytopathic effects observed during HSV-1 encephalitis may not be simply due to viral replication, but may result from additional host-mediated secondary mechanisms involving the over activation of microglial cells.

Microglial cells express a myriad of receptors from the Toll-like receptor family (TLR) of pathogen recognition receptors, including TLR2. TLR2^-/- ^mice are less susceptible to HSV infection, highlighting the importance of this receptor in the outcome of HSV encephalitis [9,10]. Using a purified murine microglial cell culture system our laboratory has shown that HSV-1 recognition through TLR2 plays a pivotal role in the initial inflammatory response associated with viral infection including the production of proinflammatory cytokines and chemokines, as well as the induction of apoptosis [5,11-13]. In other models, activation of TLR receptors has also been linked to ROS production [14-16]. Data from these systems suggests a potential role for TLR2 in the harmful effects of microglial cell-induced oxidative stress during herpes encephalitis.

In this study, we tested the hypothesis that HSV-induced neural cell oxidative tissue damage and cytotoxicity are mediated by microglial cell ROS through a TLR2-dependent mechanism. We detected elevated intracellular ROS in HSV-infected microglia obtained from wild-type mice. In contrast, the virus failed to induce ROS in microglia obtained from TLR2^-/- ^mice. Additionally, compared to wild-type microglia, TLR2^-/- ^microglia displayed attenuated HSV-induced lipid peroxidation and neurotoxicity. These studies demonstrate the importance of microglial cell TLR2 in inducing oxidative stress and neuronal damage in response to viral infection.

## Methods

### Neural cell cultures

Neural cell cultures were established from C57B/6, TLR2^-/- ^and transgenic β-actin promoter-luciferase Balb/c mice. Following dispersion of fetal (d 15 of gestation) cerebral cortices with trypsin, cells (5 × 10^5 ^or 2 × 10^5^/500 ml) were plated into collagen coated wells of 24-well plates with DMEM containing 10% heat-inactivated fetal bovine serum (FBS) and antibiotics. On day 5, the culture medium was replaced with DMEM containing 10% heat-inactivated FBS, uridine (33.6 mg/ml), and fluorodeoxyuridine (13.6 mg/ml) to suppress glial cell growth. After 24 h, cells were replaced with DMEM containing 10% heat-inactivated FBS. Culture medium was changed every 4 days thereafter. On day 7 the neural cell cultures consisted of approximately 85-90% neurons (stained with a rabbit anti-MAP-2 antibody (Chemicon; Temecula, CA) and containing characteristic processes and birefringent cell bodies), 10-15% astrocytes (stained with rabbit anti-glial fibrillary acid protein antibody; Incstar, Stillwater, MN), and < 2% microglia (stained with a rat anti-MAC-1 antibody; Roche Applied Science, Indianapolis, IN).

### Microglial cell cultures

Murine cerebral cortical cells from 1-day-old C57B/6 and TLR2^-/-^, mice were dissociated after a 30- min trypsinization (0.25%) and were plated in 75-cm^2 ^Falcon culture flasks in DMEM containing 10% heat-inactivated FBS and antibiotics. The medium was replenished 1 and 4 days after plating. On day 12 of culture harvested cells were plated in a 60-mm petri dish and incubated for 15 min at 37°C. After extensive washing with culture medium, adherent cells (microglia) were collected with a rubber policeman and centrifuged at 1000 rpm for 10 min. Purified microglial cell cultures were comprised of a cell population in which > 98% stained positively with MAC-1 antibodies and < 2% stained positively with antibodies specific to glial fibrillary acid protein, an astrocyte marker.

### Virus

HSV-1 strain 17 syn + was propagated and titrated using plaque assay on rabbit skin fibroblasts (CCL68; American Type Culture Collection, Manassas, VA).

### Intracellular reactive oxygen species assay

The production of intracellular ROS was measured by 2',7'-Dichlorofluorescin diacetate (DCFH-DA; Sigma; St. Louis, MO) oxidation. Murine microglial cultures seeded (4 × 10^4^/well) in 96-well plates or chamber slides were infected with HSV-1 (MOI = 2). At designated time points, cells were washed and incubated with HBSS containing DCFH-DA (20 μM) for 30 min. After incubation, cells were excited at 485 nm and DCFH-DA fluorescence read at 530 nm emission on a fluorescence plate reader or viewed and photographed under a fluorescence microscope. Each sample was run in triplicate and sample means were normalized to their respective controls (% of control).

### 8-isoprostane assay

Purified murine microglia from wild-type and TLR2^-/- ^mice were infected with HSV-1 (2 MOI). Cells were harvested at 90 min p.i. and 5 × 10^4 ^or 1 × 10^5 ^microglia were transferred onto mixed neural cultures (5 × 10^5^/well) from C57B/6 or TLR2^-/- ^mice creating microglia to neuron ratios of 1:10 and 1:5, respectively. To free the transferred microglia of residual HSV-1, the microglia were treated prior to harvest with 0.05% trypsin for 10 minutes, followed by 3 washes with 10% FBS containing DMEM. Transfer of 5 × 10^4 ^or 1 × 10^5 ^uninfected microglia served as a control. Oxidative damage to lipids was quantified by using an 8-isoprostane EIA kit according to the manufacturer's instructions (Cayman; Ann Arbor, MI).

### Neurotoxicity assay

Purified murine microglia from wild-type and TLR2^-/- ^mice were infected with HSV-1 (2 MOI). Cells were harvested at 90 min p.i. and transferred, at the designated microglia to neuron ratios (1:5, 1:20 and 1:40), onto cultured neurons obtained from β-actin promoter-luciferase transgenic mice. D-Luciferin potassium salt (Gold Biotechnology; St. Louis, MO) was added to each well and luciferase activity measured on a plate reader 48 h after microglial cell transfer.

### Western Blot

Microglia (2 × 10^6^/well) were infected with HSV-1 (MOI = 2). Thirty minutes following HSV-1 treatment cell lysates were collected, electrophorezed in 12% acrylamide/bis-acrylamide electrotransfered onto nitrocellulose membrane and probed with rabbit anti-phospho-p38 MAP Kinase (Thr180/Tyr182) and rabbit anti-phospho-p44/42 MAP Kinase (Thr202/Tyr204) antibodies (Cell Signaling; Danvers, MA). Rabbit anti-p38 MAP Kinase and rabbit anti-p42/p44 MAP kinase antibodies were used to detect total p38 and p42/p44 levels (Cell Signaling; Danvers, MA). Alkaline phosphatase-conjugated secondary antibodies with chemiluminescence detection was used with a Kodak Image Station to capture protein band images.

### Statistical analysis

For comparison of means of multiple groups, analysis of variance (ANOVA) was performed followed by either Fisher's protected least significant difference (PLSD)-test or Scheffe-test. For comparison of means of pairs of data a two-tailed Student's T-test for paired samples was applied.

## Results

### TLR2^-/- ^microglial cells have blunted ROS production in response to HSV-1 infection

**Figure 1:**
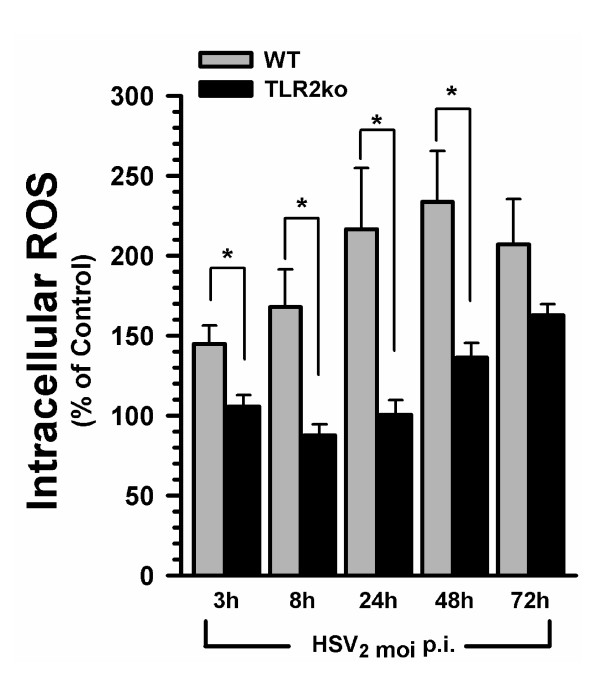
**HSV-1-infected TLR2^-/- ^microglial cells have attenuated intracellular ROS production compared to wild-type microglial cells**. Microglial cell cultures from wild type and TLR2^-/- ^mice were infected with HSV-1 (MOI = 2) and measured, at various time points, for ROS production. DCFH-DA (20 μM) was added 30 min before each time point and measured with a fluorescence plate reader. Data are normalized to ROS production in the uninfected control microglia and presented as the % of control ± SD and are representative of three separate experiments. * = p < 0.05.

To determine the role of microglia and TLR2 on HSV-1 induced oxidative damage, we first examined ROS production in wild-type microglia following HSV-1 infection *in vitro*. Purified microglial cell cultures from wild-type mice were inoculated with HSV-1 (MOI = 2), and at designated times following infection, cells were loaded with the ROS fluorescence indicator DCFH-DA. A kinetic analysis of microglial ROS production following HSV-1 challenge revealed increased intracellular ROS production as early as 3 h.p.i. ROS continued to increase at 8 h and 24 h, reaching peak ROS accumulation by 48 h.p.i. (Figure 1). In contrast, levels of ROS obtained from TLR2^-/- ^microglia did not differ from uninfected controls at 3 h, 8 h and 24 h.p.i. However, at 48 h and 72 h, increased intracellular ROS accumulation was detected in HSV-infected TLR2^-/- ^microglia. Comparison of HSV-infected wild-type and TLR2^-/- ^microglial ROS, normalized and expressed as % of their respective uninfected controls, revealed the blunted production of ROS by TLR2^-/- ^microglia. ROS production in HSV-infected TLR2^-/- ^microglia was significantly less than wild-type HSV-infected microglial at 3 h, (p = 0.043), 8 h (p = 0.026), 24 h (p = 0.022), and 48 h.p.i. (p = 0.042). These data clearly demonstrate the importance of TLR2 in HSV-induced microglial ROS production.

### Mixed neural cultures from wild-type, but not TLR2^-/- ^mice, produce oxidative damage in response to HSV-1 infection

**Figure 2:**
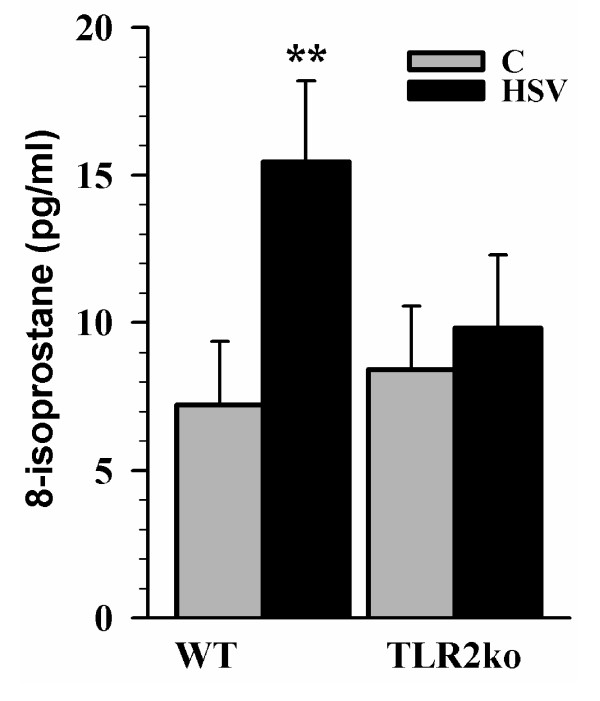
**Elevated 8-isoprostane levels in mixed neural cell cultures following HSV-1 infection**. Mixed neural cell cultures obtained from wild-type or TLR2^-/- ^mice were infected with HSV-1 (MOI = 1) for 48 h prior to collecting supernatants for 8-isoprostane assay as an indicator of lipid peroxidation. Data are presented as mean pg/mL of 8-isoprostane ± SD and are a representative of three separate experiments. ** p < 0.01.

Using a mixed neural cell culture model, we investigated the extent to which HSV-1 infection induces neuronal oxidative damage *in vitro*. HSV-1 (MOI = 1) was added to C57B/6 or TLR2^-/- ^mixed neural cell cultures (comprised of ~90% neurons, ~10% astrocytes and < 2% microglia) and the infected cultures were assessed for 8-isoprostane levels, a marker of lipid peroxidation. Based on the peak HSV-1-induced ROS production in microglial cells demonstrated above, we chose to assess oxidative damage at 48 h.p.i. HSV-1 infection was found to increase 8-isoprostane levels in wild-type mixed neural cultures (p = 0.002). In contrast, 8-isoprostane levels in TLR2^-/- ^neural cultures did not differ from untreated controls (p = 0.40), demonstrating the importance of TLR2 in HSV-1 induced oxidative damage (Figure 2). Because microglia are the TLR2 possessing cells in the brain [17], the lack of 8-isoprostane in TLR2^-/- ^cultures also suggests that oxidative damage due to direct HSV-1 infection of neurons and astrocytes is negligible, although these experiments cannot fully discount ROS production and its associated oxidative damage by astrocytes and neurons.

### TLR2 mediates microglial cell HSV-1 induced oxidative damage to mixed neural cultures

**Figure 3:**
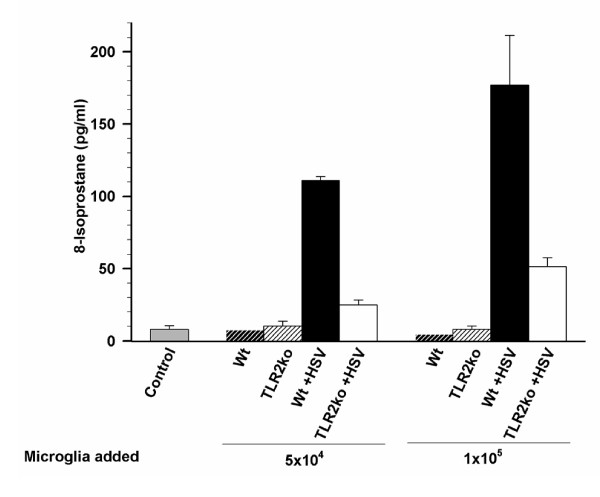
**HSV-infected microglial cells from TLR2^-/- ^mice produce less neural oxidative damage than wild-type microglia when added to mixed brain cultures**. Control or HSV-infected (MOI = 2) purified murine microglia from wild-type and TLR2^-/- ^were transferred at concentrations of 5 × 10^4 ^or 1 × 10^5 ^to wells containing a mixed neural culture. Cultures were harvested 48 h after microglial cell transfer and assessed for 8-isoprostane production. Data are presented as mean pg/mL of 8-isoprostane ± SD and are a representative of three separate experiments.

To investigate the importance of microglia in mediating HSV-induced oxidative damage, we combined our mixed neural cultures with HSV-infected or control microglial cells in reconstituted cultures. In all of these experiments, mixed neural cultures were derived from TLR2^-/- ^mice because TLR2^-/- ^mixed neural cultures have a lower background level of 8-isoprostane than wild-type cultures and are more suitable to assess the relative contribution of microglia in virus-induced oxidative damage. In brief, purified microglia cultures were infected with HSV-1 for 2 h prior to harvest and their subsequent addition to mixed neural cultures. 8-isoprostane levels were measured at 36 h.p.i. If microglia contribute to 8-isoprostane production in mixed neural cultures then the addition of previously HSV-infected microglia to naïve mixed neural cultures would increase 8-isoprostane levels. Indeed, while uninfected wild-type and TLR2^-/- ^microglia failed to increase 8-isoprostane levels above those observed in mixed neural cultures alone, the addition of HSV-infected wild-type microglia to mixed neural cultures increased 8-isoprosane levels in a dose-dependant manner (Figure 3). In comparison to HSV-infected wild-type microglia, addition HSV-infected TLR2^-/- ^microglia resulted in the blunted production of 8-isoprostane in reconstituted cultures at 5 × 10^4 ^microglia and 1 × 10^5^. Addition of HSV-infected TLR2^-/- ^microglia to mixed neural cultures did induce a marked increase in 8-isoprostane over the addition of uninfected control microglia at a density of 1 × 10^5 ^but not 5 × 10^4^.

### HSV-1 infected microglia from TLR2^-/- ^mice produce less neurotoxicity when added to mixed neural cultures

**Figure 4:**
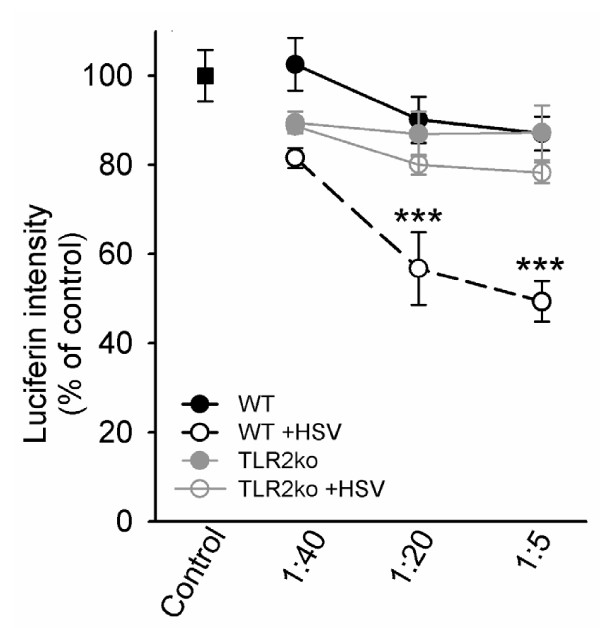
**Decreased neurotoxicity from HSV-infected TLR2^-/- ^microglia compared to wild-type cells**. Purified murine microglia from wild-type and TLR2^-/- ^mice were infected with HSV-1 (2 MOI). Cells were harvested at 90 min p.i. and transferred, at the designated microglia: neuron ratios, onto β-actin luciferase mixed neural cultures. Luciferin emission was measured 48 h after microglial cell transfer. Data were normalized to untouched β-actin luciferase mixed neural cultures and presented as the % of control. Data are presented as mean pg/mL of 8-isoprostane ± SD and are a representative of three separate experiments. *** p < 0.001.

Using neural cultures derived from β-actin promoter-luciferase transgenic Balb/c mice, we were able to assess neural cytotoxicity resulting from the addition of exogenously added HSV-infected C57B/6 microglia. In this assay, HSV-infected or control purified microglial cells were added at specific ratios to mixed neural cultures (microglia to neuron; 1:40, 1:20, 1:5) and luciferin emission measurements from each experimental group was normalized to luciferin readings from untouched mixed neural cultures (expressed as % of control). Because β-actin promoter-luciferase transgenic mice ubiquitously express the luciferase enzyme, in these studies, a decrease in luciferin intensity indicated a decrease in neural cell number. Addition of HSV-infected wild-type C57B/6 microglia resulted in a significant decrease in neural culture luciferin emission at ratios of 1:20 (p < 0.001) and 1:5 (p < 0.001) indicating increased neurocytotoxicity (Figure 4). The cytotoxicity appears to be dependent on microglia as evidenced by a more pronounced dose-dependent decrease in luciferin emission with the addition of more microglia. In contrast, addition of HSV-infected TLR2^-/- ^microglia failed to significantly decrease luciferin emission at 1:40 (p = 0.759), 1:20 (p = 0.067), and 1:5 (p = 0.052) microglia:neuron ratios and, hence, induced less cytotoxicity. Interestingly, the addition of C57B/6 background microglia to the non-syngeneic Balb/c background mixed neuronal cultures was not problematic in these experiments as evidenced by negligible neurotoxicity upon addition of uninfected C57B/6 microglia to Balb/c luciferase transgenic mixed neural cultures.

### TLR2^-/- ^microglial cells have reduced phosphorylated p38 and p42/p44 in response to HSV-1

**Figure 5:**
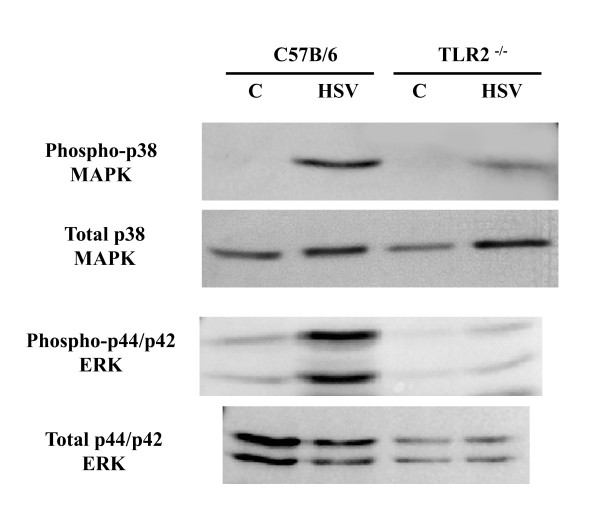
**HSV-induced p38 MAPK and p42/p44 ERK1/2 activation in primary microglia is attenuated TLR2^-/- ^mice**. Microglial cell cultures from wild-type and TLR2^-/- ^mice were infected with HSV-1 (MOI = 2) for 30 min. Cell lysates were collected and probed for total and phosphorylated p38 MAPK and p42/p44 ERK1/2.

Our laboratory has found that HSV-1 signals through TLR2 to activate NF-κB with a subsequent increase in inflammatory mediator production [11,13,17]. Microglial cell TLR2 can signal through alternative transduction cascades including p38 MAPK and p44/p42 ERK1/2, which have been implicated in free radial production [14,15,18,19]. To further investigate the role of TLR2 signaling in HSV-induced ROS production we investigated the ability of HSV-1 to activate TLR2 downstream signal transduction cascades. Addition of HSV-1 (MOI = 2) to wild-type microglia resulted in increased phosphorylation of p38 MAPK and p42/p44 ERK1/2 as detected by western blot. In contrast, a blunted p38 and p42/p44 phosphorylation response was observed using HSV-infected TLR2^-/- ^microglia (Figure 5).

## Discussion

Herpes virus brain infection results in devastating encephalitis from which, while drug therapies that inhibit viral replication have succeeded in reducing mortality, fewer than 20% of patients surviving herpes encephalitis recover without significant long-term neuropathological manifestations [1,20]. The mechanisms responsible for these long-term neurological sequelae appear to involve both viral-mediated damage and immune-mediated processes. Using a murine model, our laboratory has detailed the inflammatory and pathological time course of HSV-1 brain infection including identification of a robust and prolonged microglial cell response [1,21]. In this study, we show that HSV-induced ROS production in microglia, the resident brain macrophage, is responsible for lipid peroxidation and neurotoxicity *in vitro*. Furthermore, we show that initial microglial ROS production during HSV infection is mediated through TLR2.

Viral brain infection initiates a robust inflammatory response pivoting on the production of cytokines and chemokines by microglial cells [5]. The primary service of microglial cell activation is to combat and control invading pathogens via release of chemokines and cytokines, and subsequent recruitment of peripheral immune cells. Another key strategy is the production of ROS to damage and control the pathogen. However, excessive and prolonged ROS production and inflammatory responses may be detrimental to sensitive host cells (i.e., uninfected bystander neural cells). In the present study, we found that HSV-infected wild-type microglia robustly produced ROS with peak production of ROS at 48 h.p.i. (Figure 1). The kinetics of microglial ROS production reported in this study coincides with our previous publications reporting that peak cytokine and chemokine release from HSV-1 infected microglia occurs after 24 h.p.i. [5,11]. ROS production can dramatically impact the proinflammatory response of the inflammatory cell. Published studies show that intracellular creation of free radicals by NAPDPH oxidase drives proinflammatory cytokine production in macrophages and microglia in response to mycobacterium tuberculosis infection [15,22]. Furthermore, microglial ROS production through NADPH oxidase has been shown to precede cytokine and chemokine production [23-25].

The role of free radical-induced tissue injury has been investigated during herpes encephalitis by detection of F2-isoprotanes and F4-neuroprostanes, indicators of oxidative lipid damage [6]. We corroborate this finding *in vitro *using our mixed neural culture system where we detected increased lipid peroxidation in response to HSV-1 infection (Figure 2). Previous studies have also found that oxidative tissue damage is related to microglial ROS production in models using lipopolysaccharide (LPS) and HIV-tat protein stimulation [26-28]. In this study, we found a dose-dependent induction of 8-isoprostane in mixed neural cultures following the addition of HSV-infected microglia from wild-type mice (Figure 3). Using a similar study design, we found that microglia also contribute to neurotoxicity during HSV-1 infection (Figure 4). This finding, together with HSV-1-induced ROS production in microglia and lipid peroxidation in mixed neural cultures, demonstrates the importance of microglial cell ROS production in damage to neurons and provides evidence for the harmful effects of excess ROS accumulation.

These experiments implicate microglial cells as the primary mediator of HSV-1 induced oxidative damage. While we cannot discount the possible contribution of free radical production from neurons and astrocytes in elevating 8-isoprostane levels, the finding that proinflammatory oxidative stress during HSV-infection induced oxidative damage (Figure 3) and is neurotoxic (Figure 4) is important and may be broadly applied to other models of viral brain infection.

TLR2 is an important pathogen recognition receptor during HSV infection. Other laboratories have shown that TLR2^-/- ^mice are less susceptible to HSV-1, showing lower mortality rates following viral infection [9]. In this study, we found that HSV-induction of ROS is mediated largely through TLR2, as indicated by the lack of ROS accumulation in HSV-infected TLR2^-/- ^microglia. Furthermore, TLR2^-/- ^microglia had an attenuated ability to induce HSV-infected microglial cell-mediated oxidative tissue damage and neurotoxicity. However, lack of TLR2 did not completely prevent the production of ROS, 8-isoprostane and cytotoxicity in response to viral challenge. Recognition of HSV through alternative pattern recognition receptors, whose mRNAs are ubiquitously expressed in microglia, could provide an alternative route of viral recognition [17]. TLR3, recognizing dsRNA, and TLR9, recognizing high CpG motif carrying bacterial and viral DNAs, are likely candidates and have been shown to respond to HSV [29,30]. Another mechanism of cytokine release in macrophages is through the recognition of HSV-1 glycoprotein upon viral entry [31]. This occurs in a TLR2-independent and nucleic acid-independent manner. Based on data reported in this study, TLR2 remains a pivotal primary trigger for the downstream effects of HSV infection.

The canonical TLR signaling cascade after pathogen recognition leads to the activation of NF-κB and the subsequent production of proinflammatory mediators [11,12,17]. In this study we show for the first time that HSV-infection of microglia stimulates the MAPK signal transduction cascade via elevating levels of phosphorylated p38 MAPK and p42/44 ERK1/2 (Figure 5). p38 MAPK and p44/p42 ERK1/2 phosphorylation are commonly associated with TLR signaling and have be implicated in TLR induced ROS production [14,15,18,19]. In a similar study, Yang et al. found that monocyte production of ROS following exposure to tuberculin purified protein preceded proinflammatory mediator production and required TLR2 and p38 MAPK phosphorylation [18].

In the present study, we focused on the oxidative stress response of microglial cells during HSV-1 infection and the potential consequences of microglial ROS on neurons. In addition, we demonstrate signaling through TLR2 as a pivotal player in HSV-1 induced microglial cell ROS production. Thus, inhibition of TLR2 signalling as well as modulation of microglial cell ROS production, via an increase in antioxidative stress proteins (e.g., glutathione peroxidase-1, heme oxygenase-1), may be potential targets in regulating unwanted bystander damage to healthy neurons during viral brain infection.

## Competing interests

The authors declare that they have no competing interests.

## Authors' contributions

SJS designed and performed experiments, and drafted the manuscript. SH performed and designed experiments, and co-conceived of the study. MRL performed experiments. JRL co-conceived of the study, participated in its design and coordination, and helped draft the manuscript. All authors have read and approved the final version of this manuscript.
